# Comparison of analyses of the QTLMAS XIV common dataset. II: QTL analysis

**DOI:** 10.1186/1753-6561-5-S3-S2

**Published:** 2011-05-27

**Authors:** Sebastian Mucha, Marcin Pszczoła, Tomasz Strabel, Anna Wolc, Paulina Paczyńska, Maciej Szydlowski

**Affiliations:** 1 Department of Genetics and Animal Breeding, Poznan University of Life Sciences, Wolynska 33, 60-637 Poznan, Poland; 2 Animal Breeding and Genomics Centre, Wageningen UR Livestock Research, 8200 AB Lelystad, The Netherlands; 3 Animal Breeding and Genomics Centre, Wageningen Institute of Animal Sciences Wageningen University, 6700 AH Wageningen, The Netherlands; 4 Department of Animal Science and Center for Integrated Animal Genomics, Iowa State University, Ames, IA 50011-3150, USA

## Abstract

**Background:**

A quantitative and a binary trait for the 14^th^ QTLMAS 2010 workshop were simulated under a model which combined additive inheritance, epistasis and imprinting. This paper aimed to compare results submitted by the participants of the workshop.

**Methods:**

The results were compared according to three criteria: the success rate (ratio of mapped QTL to the total number of simulated QTL), and the error rate (ratio of false positives to the number of reported positions), and mean distance between a true mapped QTL and the nearest submitted position.

**Results:**

Seven groups submitted results for the quantitative trait and five for the binary trait. Among the 37 simulated QTL 17 remained undetected. Success rate ranged from 0.05 to 0.43, error rate was between 0.00 and 0.92, and the mean distance ranged from 0.26 to 0.77 Mb.

**Conclusions:**

Our comparison shows that differences among methods used by the participants increases with the complexity of genetic architecture. It was particularly visible for the quantitative trait which was determined partly by non-additive QTL. Furthermore, an imprinted QTL with a large effect may remain undetected if the applied model tests only for Mendelian genes.

## Background

Genome-wide association studies show that for some traits markers with estimated significant effects explain little of the genetic variability estimated from the population studies, so called missing heritability problem [1]. This phenomenon may be partially explained by rare combinations of common variants (epistasis). Recent studies in livestock contribute new evidence on the important role of gene interactions [2]. Although high density genotyping without implemented biomolecular networks is unlikely to discover much of epistasis across the genome, there is hope that it may detect some of the effects, which are tagged by available marker SNPs. Furthermore, some variants add to a phenotype only when inherited from a specific parent. Recent reports indicate that such parent-of-origin effects (imprinting) may contribute more than 10% of the heritability of complex traits [3]. Even if imprinted regions are discovered their effect would be biased under models that do not take the sex-specific association into account.

Common dataset for QTLMAS 2010 workshop was simulated under a model which combined additive inheritance, epistasis and imprinting[4]. Here, we compare results submitted by the participants of the 14^th^ QTLMAS Workshop. In total seven groups reported QTL for the simulated quantitative trait and five of them reported QTL for the simulated binary trait.

## Methods

### Simulated data

The simulated data set was described by Szydłowski and Paczyńska [4]. Shortly, the pedigree consisted of 2326 phenotyped individuals in 4 generations descending from 5 male and 15 female founders Females were mated once and gave birth to about 30 progeny. Genome consisted of 5 approximately 100mln bp long chromosomes. Two genetically correlated traits were simulated, a quantitative trait and a binary trait. The quantitative trait was determined by 30 additive QTL located on chromosomes 1–4, 2 pairs of epistatic QTL located on chromosomes 1–2, and 3 maternally imprinted QTL located on chromosome 2. The binary trait was affected only by a subset of 22 additive QTL determining the quantitative trait. The QTL differed in the percentage of explained genetic variance. There were many QTL with small effect on chromosome 4, and a group of QTL with intermediate effects on chromosomes 1 and 2. The two major QTL were located on chromosome 3, whereas chromosome 5 contained no QTL. Each individual was genotyped for 10031 biallelic SNPs, however, the genotypes were unordered, thus it was unknown which allele originated from which parent. Average LD (r^2^) between adjacent SNPs was 0.1. Each simulated QTL was surrounded by 19-47 SNPs located within 1Mb distance from the QTL.

### Variance explained by the QTL

Participants reported QTL positions along with variance explained by the QTL. For the quantitative trait, variance of a QTL was expressed as a genetic variance or a percentage of genetic variance, whereas for the binary trait variance was expressed as a percentage of genetic variance or it was reported on an arbitrary scale.

### Methods used by the participants

A variety of methods was used to analyze the common dataset. The Bayesian models used by the participants, differ in the way they shrink the effects of individual SNPs. Sun et al. [5] applied BayesCpi, in which all SNP effects have a common variance and the probability that a SNP has zero effect is treated as unknown with a uniform prior. Bouwman et al. [6] used BVSM (Bayesian variable selection method) with a mixture of two normal distributions with some fixed variances and a priori 5% chance for an SNP to be a QTL. Calus et al. [7] used BayesC, which also uses a mixture of distributions for small and large SNP effects, and considered a value of 50 as the expected number of QTL.

Some authors considered alternative models and less heavy computations. Coster and Calus [8] analyzed one chromosome at a time with partial least squares regression (PLSR), which is based on principal component analysis. Shen et al. [9] applied double hierarchical generalized linear models (DHGLM), which allows for marker-specific variances. Karacaören et al. [10] used a GRAMMAR (genome-wide rapid association using mixed model and principal components analyses) to account for family effect and linkage disequilibrium. Nettelblad [11] utilized the pedigree information and multiple markers to estimate probabilities for all 40 founder alleles at a locus and then used forward selection to find QTL.

Various criteria were applied to test for the presence of a QTL. Bouwman et al. [6] used threshold of 3.2 for the parameter-wise Bayes Factor. Calus et al. [7] obtain significance threshold from permutation of genotypes against the phenotype and pedigree data. Also Nettelblad [11] derived the significance threshold from random permutation. Coster and Calus [8] estimated a smooth curve through the standardized regression coefficients of all markers and considered its local maxima as QTL. Shen et al. [9] searched for QTL which had variance greater than the overall variance estimate from GLMM. Sun et al. [5] considered 3 strategies to calculate the threshold: permutation of phenotypes against genotypes, random simulation of SNP genotypes of individuals in the first 4 generations using the pedigree and SNP placement from the QTL-MAS 2010, and simulation of LD between the simulated SNPs in the founder generation at a level similar to that found in the original data.

### Comparison criteria

Since a dense genetic map was available (average marker spacing approx. 50 kb), a true QTL was considered mapped if one or more of the submitted positions were within 1 Mb distance from the QTL. Sometimes one submitted position mapped two different QTL, therefore quite often the number of mapped positions exceeded the number of reported positions. If two submitted positions were within 1 Mb distance from a simulated QTL, they were considered to map the same true QTL. Reported positions were considered as false positives, if a distance to the closest true QTL exceeded 1 Mb.

Two criteria were used to compare the results submitted by the participants: the success rate (ratio of mapped QTL to the total number of simulated QTL), and the error rate (ratio of false positives to the number of reported positions). Mean distance between a true mapped QTL and the nearest submitted position was calculated to compare the precision of applied methods.

Participants used different scales to describe the variance contributed by the mapped QTL. Where possible, we transformed each submitted variance to the percentage of true additive variance for QT and calculated the sum of the individual variances contribued by QTL mapped by the participants.

## Results

### Additive QTL

**Figure 1:**
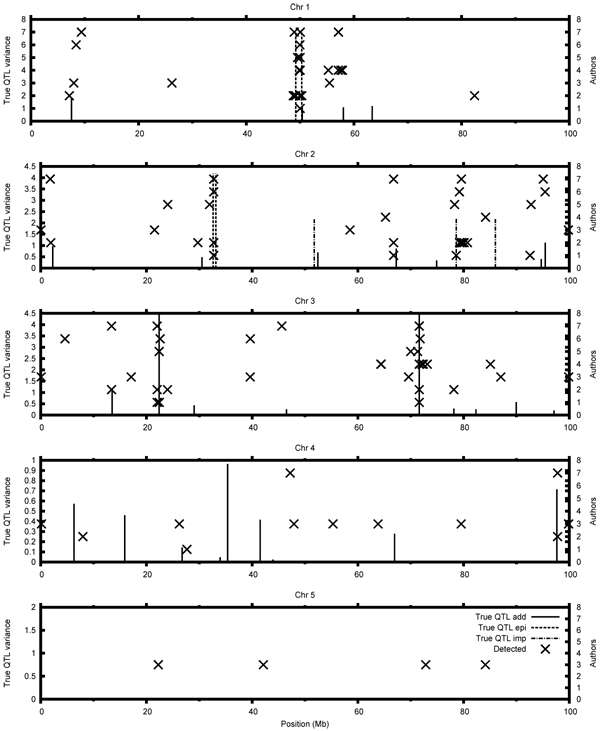
**Comparison of results for the quantitative trait. **Where 1 – Bouwman et al., 2 – Calus et al., 3 – Coster and Calus, 4 – Karacaören et al., 5 – Nettelblad, 6 – Shen et al., 7 – Sun and Dekkers.

Most of the participants managed to map the two major QTL on chromosome 3 (Figure 1), especially the QTL at the position 71.6 Mb, which was easy to map since the observed marker had a direct effect on the trait. The second major QTL at the position 22.4 Mb could have been mapped utilizing LD with surrounding markers. The remaining additive QTL on chromosomes 1-3 were quite often detected by the participants. However, small QTL on chromosome 4 had been hard to detect and only two of them (positions 26.7 Mb and 97.7 Mb) were found. Even though there was no QTL on chromosome 5, some groups found associations between the markers on this chromosome and phenotypes (i.e. PLSR yielded many false positives). Therefore, chromosome 5 can be considered as a check for false positives.

**Figure 2:**
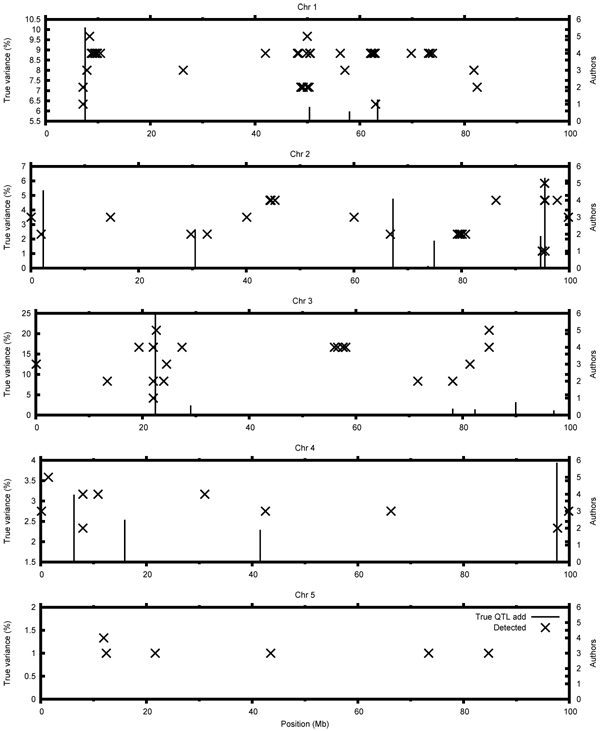
**Comparison of results for the binary trait**.Where: 1 – Bouwman et al., 2 – Calus et al., 3 – Coster and Calus, 4 – Karacaören et al., 5 – Shen et al.,

For the quantitative trait, the highest number of correctly mapped QTL was reported by Sun et al. [5], who used BayesCpi. Other Bayesian methods used by Bouwman et al. [6] and Calus et al. [7] also resulted in a high number of detected QTL. Apart from the Bayesian methods, DHGLM [9] also performed well in the detection of QTL for the quantitative trait. The PLSR [8], GRAMMAR [10] and haplotype inference had a higher error rate along with low success rate. For the binary trait, the BayesC method used by Calus et al. [2] resulted in the highest number of correctly mapped QTL (Figure 2).

### Epistatic QTL

The interacting QTL on chromosome 1 were approx. 1.1Mb distance apart with very little LD between these 2 sites. The pair contributed approx. 7% of phenotypic variance. The two QTL located were mapped by all groups except of one. Most of the groups which found the epistatic QTL reported many positions in the adjacent regions. Two groups, which used phasing and machine learning [11,10], submitted two positions which mapped the two QTL. On the other hand, two participants [6,9] mapped the two QTL with just one submitted position.

The other pair was located on chromosome 2. The two functional SNPs were approx. 0.5Mb apart and contributed approx. 4.2% of the phenotypic variance. Due to uneven distribution of haplotypes, these epistatic effects were harder to detect. This effect was detected by 5 out of 7 groups. It is worth mentioning that the epistatic QTL were located very close to each other. That is why often one reported position mapped two QTL. Some participants found more than two QTL per epistatic region, which suggests a problem with false positives.

### Imprinted QTL

The three imprinted QTL were placed on chromosome 2, however they were independent (r^2^<0.008). Even though they all had similar effects, only the QTL located at the position 78.6 Mb was mapped correctly, whereas the remaining two QTL were undetected. All of the groups that had found the imprinted QTL underestimated its effect, possibly because the models did not account for imprinting.

### Estimated positions and variances of the QTL

Participants mapped the QTL with different precision. Mean distance between the true QTL and submitted positions were calculated for each participant. For the quantitative trait, the precision ranged from 0.26 Mb to 0.62 Mb, whereas for the binary trait the mean distance was larger and ranged from 0.30 Mb to 0.77 Mb. Most of the methods mapped QTL with a similar precision, except of PLSR [8], which was substantially less precise (Table 1, Table 2.). We observed that the variance of the mapped QTL was not highly overestimated (Table 3).

**Table 1:** Comparison of submitted results for the quantitative trait

Authors	Method	Reported positions	Mapped QTL	Mean dist. (Mb)	False positives	Success rate	Error rate
Bouwman et al.	BVSM	9	10	0.34	1	0.27	0.11
Calus et al.	BayesC	24	15	0.26	6	0.41	0.25
Coster and Calus	PLSR	25	2	0.62	20	0.05	0.92
Karacaören et al.	GRAMMAR	16	5	0.31	7	0.14	0.44
Nettelblad	Haplotype inference	10	7	0.34	3	0.19	0.30
Shen et al.	DHGLM	9	11	0.42	2	0.30	0.22
Sun et al.	BayesCPi	15	16	0.41	2	0.43	0.13

**Table 2:** Comparison of submitted results for the binary trait

Authors	Method	Reported positions	Mapped QTL	Mean dist. (Mb)	False positives	Succes rate	Error rate
Bouwman et al.	BVSM	5	5	0.30	0	0.23	0.00
Calus et al.	BayesC	24	8	0.33	14	0.36	0.58
Coster and Calus	PLSR	22	5	0.77	17	0.23	0.77
Karacaören et al.	GRAMMAR	50	5	0.33	41	0.23	0.82
Shen et al.	DHGLM	6	5	0.45	2	0.23	0.33

**Table 3:** Comparison of submitted results: additive variance explained by the mapped QTL

Participant	Mapped additive QTL (30 true QTL)	Percentage of additive variance contributed by mapped QTL	
		True	Estimated
		
Bouwman et al.	5	27.6	26.7
Calus et al.	10	47.9	54.4
Coster and Calus	2	7.7	6.8
Nettelblad			
Method A	2	21.8	24.8
Method B	3	23.7	19.1
Sun et al.			
Stringent	11	48.9	61.5
Liberal	13	58.7	62.5

### Pleiotropy

Most of the groups realized that the two traits were correlated and accounted for this fact in the analysis. Bouwman et al. [6] used a bivariate model and found pleiotropic QTL. Coster and Calus [8] performed a joint analysis of the two traits in PLSR. Calus et al. [7] used a range of models (univariate and bivariate) and obtained the best results with a bivariate BayesC model. Bivariate analysis allowed accounting for the genetic correlation between the two traits which was beneficial for the accuracy.

## Discussion

Participants of the XIVth QTL-MAS Workshop presented a wide variety of methods for the analysis of genomic data. The methods differed both with respect to complexity and calculation time. Differences between the methods were particularly visible in the analysis of the quantitative trait which had a complex genetic background. Apart from the additive QTL which were most frequent, there were some imprinted and epistatic QTL. For QT the true QTL variance was heterogeneous across the genome. For such data we observed that Bayesian models performed better than other methods. Apart from additive QTL which are most frequent, there were some imprinted and epistatic QTL with average effects. For QT the true QTL variance was heterogeneous across the genome. For such data we observed superiority of the models that for SNP effects use a mixture of two distributions.

It is worth mentioning that although the interacting QTL were detected, participants were rather unaware of the existing epistasis. The precise mapping of the two epistatic QTL pairs was difficult. The number of SNPs on simulated chromosomes was large enough to prohibit the use of the exhaustive algorithms that explicitly enumerate all possible SNP combinations. One of the heuristic approaches is a two-step approach, where first a subset of SNPs is selected according to certain criteria and they are used for subsequent search for epistatic effects. However, because in our simulation the interacting SNPs had no marginal effects, this approach would be insufficient.

Family-based designs allow testing for the parent-of-origin effects on phenotypes after the ordered genotypes are reconstructed at each marker. To fast reconstruct multiple ordered genotypes in complex pedigree one can use DSS algorithm implemented in PedPhase 3.0 [12]. Our own calculation shows that all genes were easily detected within the phenotyped population (N=2326) if individual SNPs were tested under true (maternal imprinting) model from ordered genotypes (P<1.98×10^ 7^). Furthermore, Nettelblad [11] showed that the density of SNPs and the amount of LD were sufficient to phase the censored data, therefore the recovery of allele origin was also possible. Hence, we concluded that the available censored data were sufficient to map all of the imprinted QTL. We observed, however, that the available family structure in the simulated data set was not fully utilized in the performed genome scans. Only 1 out of 3 imprinted QTL was found with models that do not take parent-of-origin effect into account. Five groups estimated the effect of this QTL, however it was strongly underestimated and ranged between 1.70 and 0.01, whereas the true effect was equal to 3.00. Our comparison shows that an imprinted QTL with a large effect may remain undetected in GWAS if an applied model tests only for Mendelian genes and does not take parent-of-origin effect into account. The hope that all major imprinted genes will be discovered with standard Mendelian models, albeit underestimated, is not fully justified.

Our comparison shows that many QTL were not detected despite of sufficient information provided in the released dataset. Furthermore, we showed that already developed models and methods were sufficient to map those genes. One reason why QTL were undetected was simply that participants paid little attention to nonmendelian gene actions and interactions. The mapping strategies were almost entirely focused on localization of Mendelian additive genes, which can be most easily utilized in selection. It is also worth mentioning that some of the methods produced a large proportion of false positives (PLSR [8], haplotype inference [11], GRAMMAR [10]). This may indicate that a better control of false positives is required to improve efficiency of those methods.

Among the 30 simulated additive Mendelian QTL for the quantitative trait, 15 were not detected by any of the applied methods. It can be partially explained by low LD with the available SNPs or their small effects.

## Conclusions

All participants were animal and plant breeders and draw their attention to additive Mendelian genes that can be utilized in selection regardless of the fact that gene location is not necessary for evaluation of genomic BV. To localize important QTL they often used the same methods that are utilized in genome-wide breeding value estimation, and therefore their results were often limited to the additive QTL. However in the future, pedigreed livestock populations under genomic selection may significantly contribute to the knowledge on complex genetic mechanisms, including non-additive and non-Mendelian inheritance. Differences among methods used by the participants were particularly visible for the quantitative trait which was determined partly by non-additive QTL. Bayesian models mapped more QTL when true genetic model was heterogeneous across the genome. This advantage was less apparent in case of the binary trait, where the true model approached a homogenous QTL variance model. Estimated variance of the detected QTL was not overestimated if models utilized all markers simultaneously (penalized estimator in Bayesian models). Our comparison shows that an imprinted QTL with a large effect may remain undetected if the applied model tests only for Mendelian genes and does not take parent of origin effect into account. Differences among methods used by the participants increased with trait complexity. Models that applied mixture of two distributions for SNP effects mapped more QTL when true genetic model was heterogeneous across the genome. This advantage disappeared when the true model approached a homogenous QTL variance model.

## Competing interests

The authors declare that they have no competing interests.

## Authors’ contributions

All authors conceived the study, contributed to methods and to writing the paper and also read and approved the final manuscript. MS and PP simulated the 14th QTLMAS Workshop data set, SM wrote the first draft. TS and MS provided overall oversight of the project.
